# Prolyl isomerase Pin1 binds to and stabilizes acetyl CoA carboxylase 1 protein, thereby supporting cancer cell proliferation

**DOI:** 10.18632/oncotarget.26691

**Published:** 2019-02-26

**Authors:** Koji Ueda, Yusuke Nakatsu, Takeshi Yamamotoya, Hiraku Ono, Yuki Inoue, Masa-Ki Inoue, Yu Mizuno, Yasuka Matsunaga, Akifumi Kushiyama, Hideyuki Sakoda, Midori Fujishiro, Shin-Ichiro Takahashi, Akio Matsubara, Tomoichiro Asano

**Affiliations:** ^1^ Department of Medical Science, Graduate School of Medicine, Hiroshima University, Hiroshima City, Hiroshima, Japan; ^2^ Department of Clinical Cell Biology, Graduate School of Medicine, Chiba University, Chuo-ku, Chiba City, Japan; ^3^ Center for Translational Research in Infection & Inflammation, School of Medicine, Tulane University, New Orleans, LA, USA; ^4^ Division of Diabetes and Metabolism, Institute for Adult Diseases, Asahi Life Foundation, Chuo-ku, Tokyo, Japan; ^5^ Division of Neurology, Respirology, Endocrinology, and Metabolism, Department of Internal Medicine, Faculty of Medicine, University of Miyazaki, Kiyotake, Miyazaki, Japan; ^6^ Division of Diabetes and Metabolic Diseases, Nihon University School of Medicine, Itabashi, Japan; ^7^ Department of Applied Biological Chemistry, Graduate School of Agricultural and Life Sciences, The University of Tokyo, Bunkyo-ku, Tokyo, Japan; ^8^ Department of Urology, Graduate School of Biomedical and Health Science, Hiroshima University, Hiroshima, Japan

**Keywords:** Pin1, ACC1, cancer metabolism

## Abstract

The prolyl isomerase Pin1 expression level is reportedly increased in most malignant tissues and correlates with poor outcomes. On the other hand, acetyl CoA carboxylase 1 (ACC1), the rate limiting enzyme of lipogenesis is also abundantly expressed in cancer cells, to satisfy the demand for the fatty acids (FAs) needed for rapid cell proliferation. We found Pin1 expression levels to correlate positively with ACC1 levels in human prostate cancers, and we focused on the relationship between Pin1 and ACC1. Notably, it was demonstrated that Pin1 associates with ACC1 but not with acetyl CoA carboxylase 2 (ACC2) in the overexpression system as well as endogenously in the prostate cancer cell line DU145. This association is mediated by the WW domain in the Pin1 and C-terminal domains of ACC1. Interestingly, Pin1 deficiency or treatment with Pin1 siRNA or the inhibitor juglone markedly reduced ACC1 protein expression without affecting its mRNA level, while Pin1 overexpression increased the ACC1 protein level. In addition, chloroquine treatment restored the levels of ACC1 protein reduced by Pin1 siRNA treatment, indicating that Pin1 suppressed ACC1 degradation through the lysosomal pathway. In brief, we have concluded that Pin1 leads to the stabilization of and increases in ACC1. Therefore, it is likely that the growth-enhancing effect of Pin1 in cancer cells is mediated at least partially by the stabilization of ACC1 protein, corresponding to the well-known potential of Pin1 inhibitors as anti-cancer drugs.

## INTRODUCTION

The morbidity of cancers is increasing, and development of novel therapies is eagerly awaited. Cancer cells have various distinguishing features contributing to their high proliferation rates, which could serve as a target for novel therapies [1]. From the perspective of metabolism, most cancer cells exhibit a marked increase in glycolysis and suppression of the oxidative phosphorylation needed to produce ATP, a phenomenon known as the Warburg effect [2–3]. Thus, cancer cells incorporate and consume far larger amounts of glucose than normal cells. Although the underlying molecular mechanism has not yet been fully elucidated, it reportedly involves increased expression of prolyl isomerase Pin1 which regulates both pyruvate kinase M2 and phosphoglycerate kinase 1 in cancer cells [4–5].

On the other hand, generation of fatty acids (FAs) is also essential for cell proliferation, since FAs are utilized as cell membrane materials, serve as an energy source and mediate signal transduction [6–8]. Among many lipid metabolism enzymes, ACC1, which converts from acetyl CoA to malonyl CoA is one of the rate limiting enzymes involved in lipogenic processes [9]. Increased production of malonyl CoA leads to suppression of carnitine palmitoyl transferase activity which is a rate limiting enzyme of FA oxidation [10]. AMPK reportedly phosphorylates and inhibits the activities of ACC1 and ACC2, which results in lipid depletion [11–12]. Furthermore, recent reports have clarified the metabolism of FAs to also play an important role in redox homeostasis and the regulation of several oncogenic signaling pathways including NANOG, Wnt/beta-catenine and Hippo/YAP [13]. Therefore, the inhibition of FA synthesis using inhibitors or genetic manipulations reportedly suppresses the growth of various cancers including prostate, ovarian and breast tumors [14–18].

In this study, we focused on the correlation between the ACC1 protein level and that of Pin1. Pin1 recognizes the phosphorylated Ser/Thr-Pro containing motif and induces cis-trans isomerization of proline [19–21]. Pin1 expression levels are markedly increased in most cancers and the degree of elevation correlates negatively with patient outcomes [22–24]. Numerous reports have demonstrated Pin1 to function as a master regulator of cancer by regulating a variety of oncogenes and tumor suppressors. For examples, Pin1 enhances the recruit of c-myc [25] or estrogen receptor [26] on DNA and promotes transcriptional activity. On the other hands, Pin1 causes the hyperphosphorylation of tumor suppressor retinoblastoma protein and inactivates. Moreover, Pin1 stabilizes the protein of cyclin D1 [27, 28] and NF-κB p65 [29], while promoting the degradation of tumor suppressor Fbw7 [30].

Therefore, we focused on the mechanism linking Pin1 and ACC1, and identified ACC1 as a direct target of Pin1. This is the first report clarifying the role of Pin1 in lipid metabolism in cancer cells, and may thus provide insights useful for developing new anti-cancer drugs [31–34].

## RESULTS

### Pin1 knockdown in prostate cancer cells decreases FA contents

**Figure 1:**
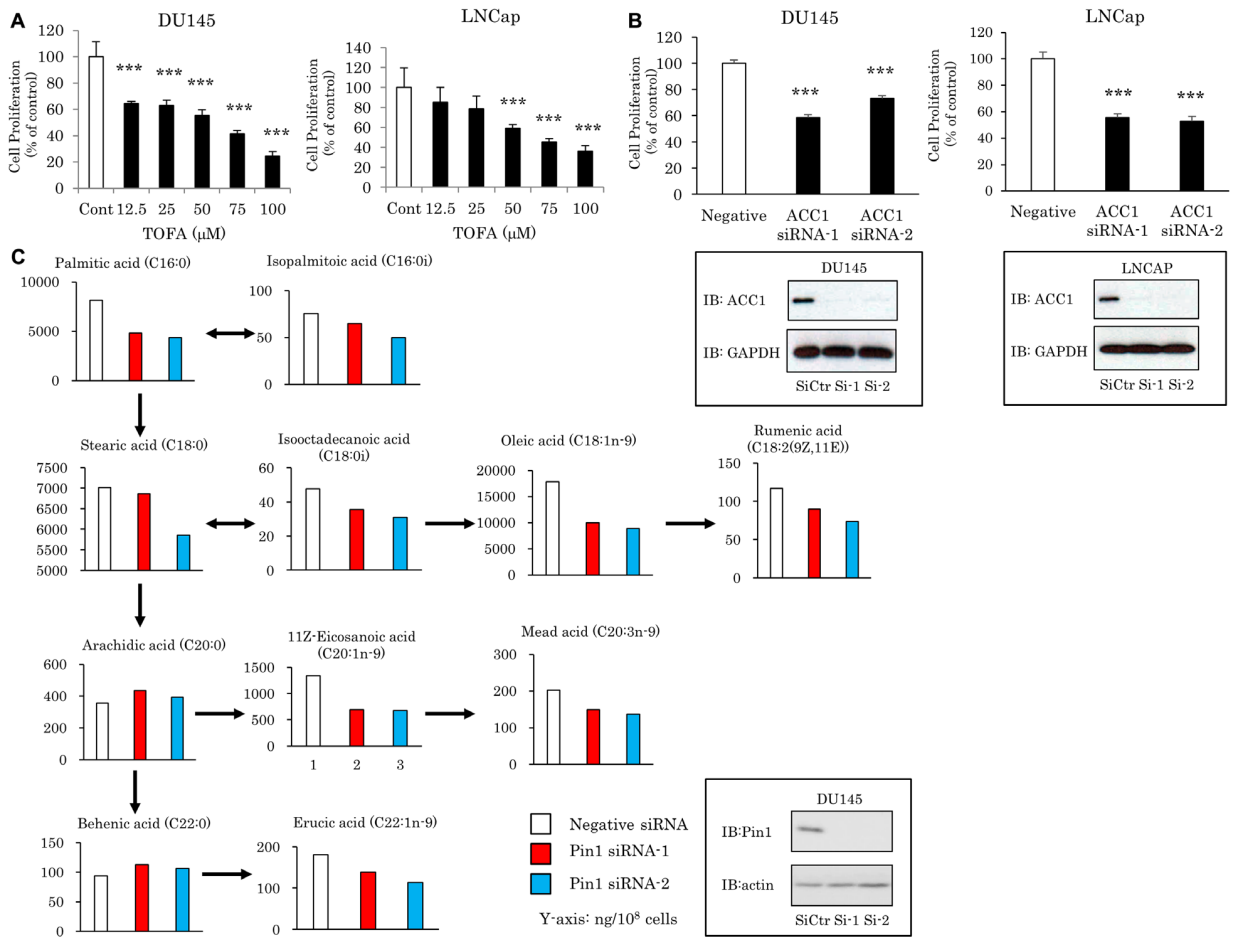
Pin1 knockdown disrupts the FA amounts in prostate cancers (**A, B**) Both DU145 and LNCap cells were treated with the ACC inhibitor TOFA or ACC1 siRNA for 48 hrs. Then, MTT assay was performed. (*n* = 4) (**C**) DU145 cells were treated with two types of Pin1 siRNA. Then, the same numbers of cells were subjected to lipidomics analysis. In the enclosure is the same condition sample blotting. ^*^P < 0.05, ^**^P < 0.01, ^***^P < 0.001.

To examine the contribution of endogenous FA synthesis to cellular proliferation, two types of prostate cancer cells, DU145 and LNCap, were treated with the ACC inhibitor 5-(tetradecyloxy)-2-furancarboxylic acid (TOFA). Treatment with TOFA clearly suppressed the growth of both DU145 and LNCap cells, in a concentration-dependent manner (Figure 1A). ACC1 knockdown by siRNA also significantly suppressed the proliferation of both cell lines (Figure 1B), indicating that disruption of the FA supply suppresses cancer cell proliferation.

On the other hand, Pin1 reportedly contributes to the malignant features of cancer cells. We thus investigated the role of Pin1 in lipid metabolism in cancer cells. Accordingly, lipidomics analysis was performed to evaluate whether Pin1 impacts FA contents in prostate cancers. It was demonstrated that siRNA-induced suppression of Pin1 significantly reduced the amounts of several FA species in DU145 cells (Figure 1C). These results suggested the commitment of Pin1 in the regulation of endogenous synthesis of FAs.

### Pin1 interacts with ACC1, but not ACC2

**Figure 2:**
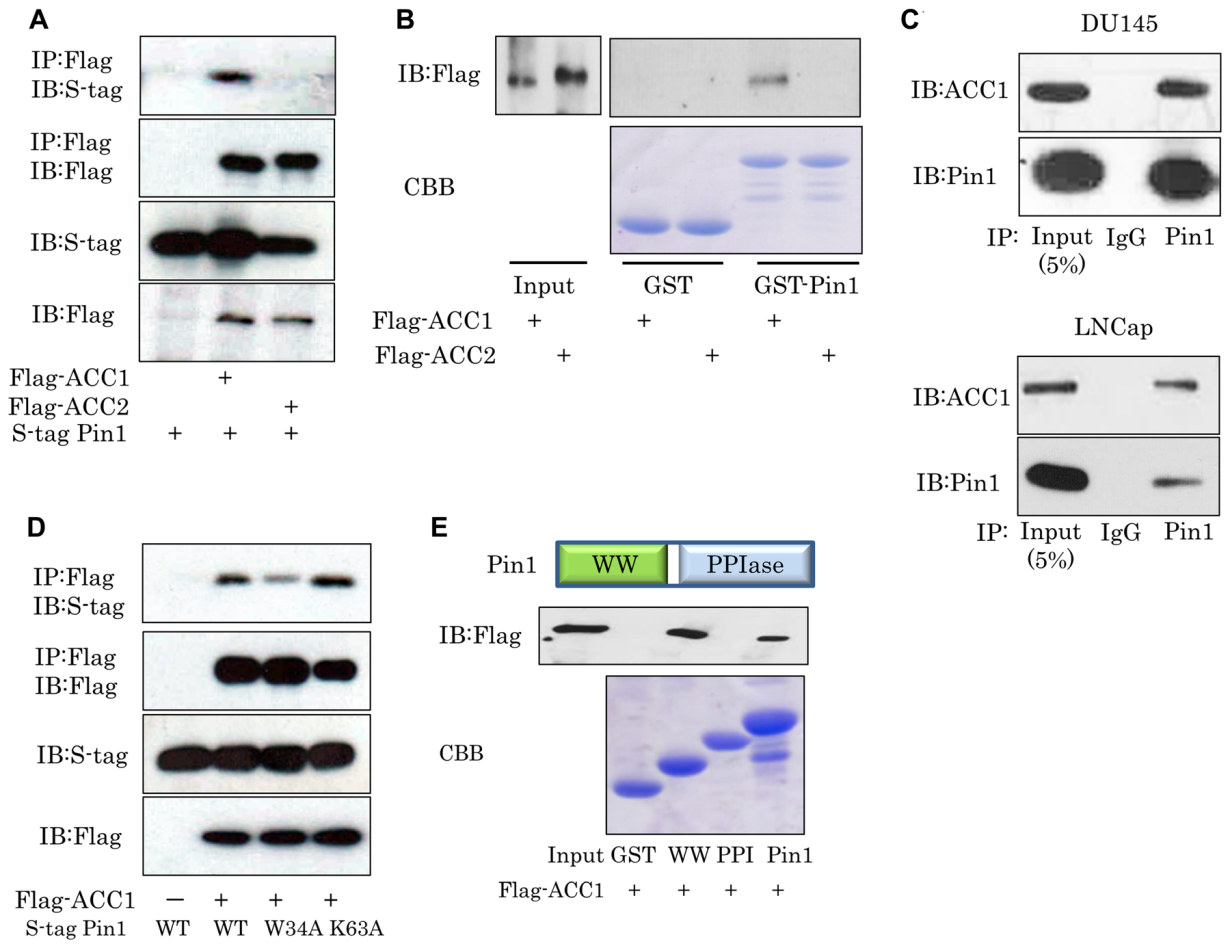
Pin1 interacts with ACC1, but not with ACC2 (**A**) S-tag Pin1 was overexpressed with Flag-ACC1 or Flag-ACC2 in HEK-293T cells. Then, immunoprecipitations were performed, using Flag beads. (**B**) Flag-ACC1 or Flag-ACC2 was transfected into HEK-293T cells. Then, lysates were prepared and were reacted with GST or GST-Pin1. (**C**) Cell lysates were prepared from DU145 or LNCap cells. Finally, immunoprecipitations were carried out with IgG control antibody or Pin1 antibody. (**D**) Flag-ACC1 was overexpressed with wild type Pin1 or Pin1 mutants in HEK-293T cells. Then, immunoprecipitations were performed. (**E**) Cell lysates containing Flag-ACC1 were reacted with GST-fused proteins.

As Pin1 knockdown reduced the amount of palmitic acid (C16:0), we speculated that Pin1 enhanced *de novo* synthesis of FAs. In lipogenesis, ACC1 and ACC2 are rate limiting enzymes and their inhibition suppresses cancer growth through the depletion of FAs. Therefore, we examined the associations between Pin1 and ACC. For this purpose, S-tagged Pin1 was co-transfected with Flag-tagged ACC1 or ACC2 into HEK-293T cells. Then, immunoprecipitations were performed. An interaction between Pin1 and ACC1 was clearly observed, while Pin1 did not interact with ACC2 (Figure 2A). Pull-down assay using GST and GST-Pin1 from the cell lysates containing Flag-tagged ACC1 or ACC2 also provided evidence of the interaction between Pin1 and ACC1 (Figure 2B). The association between endogenous Pin1 and ACC1 was demonstrated by immunoblotting with the anti-Pin1 antibody, followed by immunoprecipitations with anti-ACC1 antibody in both DU145 and LNCap cells. (Figure 2C) In contrast, no association between Pin1 and fatty acid synthase (FASN) was detected (data not shown).

Next, we investigated the association of S-tagged wild-type and two mutated Pin1 with Flag-tagged ACC1. While W34A Pin1 mutant is reportedly unable to bind to pSer/Thr-Pro containing motif, K63A Pin1 mutant retains the binding ability but lacks PPIase activity. The association of W34A Pin1 mutant with ACC1 was markedly attenuated as compared with wild-type or K63A Pin1 (Figure 2D). To determine the domain in Pin1 that associates with ACC1, cell lysates containing Flag-ACC1 were subjected to pull-down assay using GST alone, GST-full length Pin1, the GST-WW domain or the PPI domain of Pin1. WW but not the PPI domain of Pin1 was identified as being essential for binding with ACC1 (Figure 2E).

### C-terminal carboxyltransferase domain of ACC1 is essential for binding with Pin1

**Figure 3:**
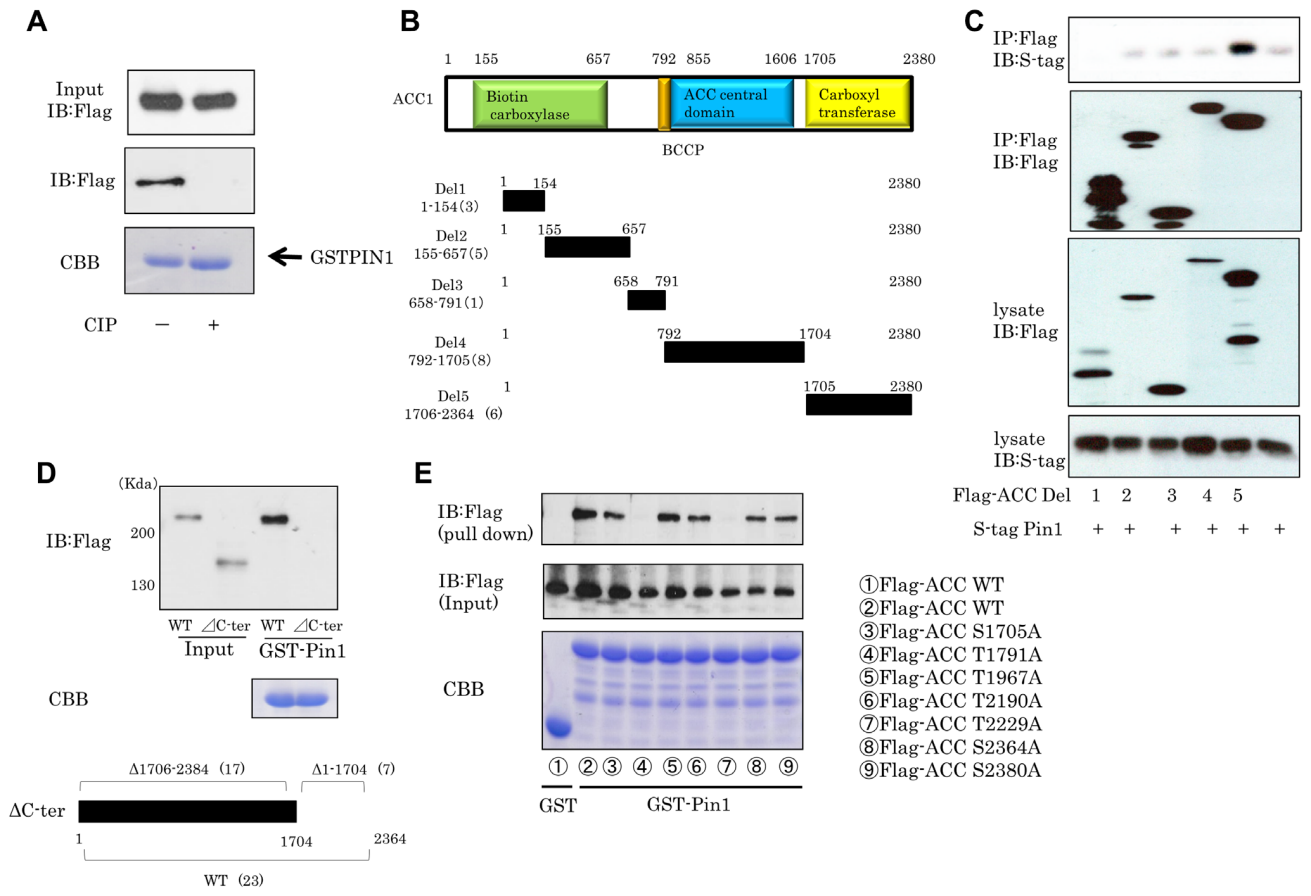
Two ACC1 sites are required for binding with Pin1 (**A**) Cell lysates containing Flag-ACC1 were immunoprecipitated with Flag beads. The beads were then incubated with CIAP for 1hr. Flag-ACC1 was eluted by Flag peptide and mixed with GST-Pin1 beads. (**B**) Five deletion mutants of ACC1 were created. (**C**) S-tag Pin1 was overexpressed with deletion mutants of ACC1 and, next, immunoprecipitations were carried out. (**D**) ACC1 mutants lacking Del5 were created. The pull-down assay was then performed. (**E**) Each Thr or Ser in seven putative Pin1 binding sites in ACC1 Del5 was substituted with Ala, and pull-down assays were performed with GST-Pin1 beads.

Since the WW domain of Pin1 reportedly recognizes and interacts with the phosphorylated Ser/Thr-Pro containing motif, it was examined whether the phosphorylation of ACC1 was required for association with Pin1. Flag-tagged ACC1 was overexpressed in HEK-293T cells and the cell lysates were treated with or without CIAP, and then subjected to the pull-down assay using GST-Pin1. It was shown that ACC1 dephosphorylated by CIAP treatment did not associate with GST-Pin1, indicating the phosphorylation of ACC1 to be essential for interacting with Pin1 (Figure 3A). Then, to narrow the candidate portions of ACC1 containing the Ser/Thr-Pro motif involved in the association with Pin1, five ACC1-deletion mutants were created (Figure 3B). Each these five Flag-ACC1 deletion mutants and S-tagged Pin1 were transfected into HEK-293T cells and immunoprecipitation experiments were then performed. These experiments revealed that the carboxyltransferase (CT) domain of ACC1 (Del 5), but not the other four constructs, associated with Pin1 (Figure 3C). Conversely, the ACC1 protein in which Del5 was deleted (∆C-terminal domain-ACC1) was unable to interact with GST-Pin1 (Figure 3D). For identification of further detailed binding sites, Thr or Ser in each of 7 Ser/Thr-Pro motifs in the CT domain were substituted with Ala and these Flag-tagged constructs were overexpressed in HEK-293T cells, and subjected to the pull-down assay with GST-Pin1. It was found that both T1791A and T2229A mutants completely failed to bind with GST-Pin1, indicating that these two Ser/Thr-Pro motif containing sites in the CT domain of ACC1 are crucial for binding with Pin1 (Figure 3E).

### Pin1 increases the protein amount of ACC1 without affecting mRNA level

**Figure 4:**
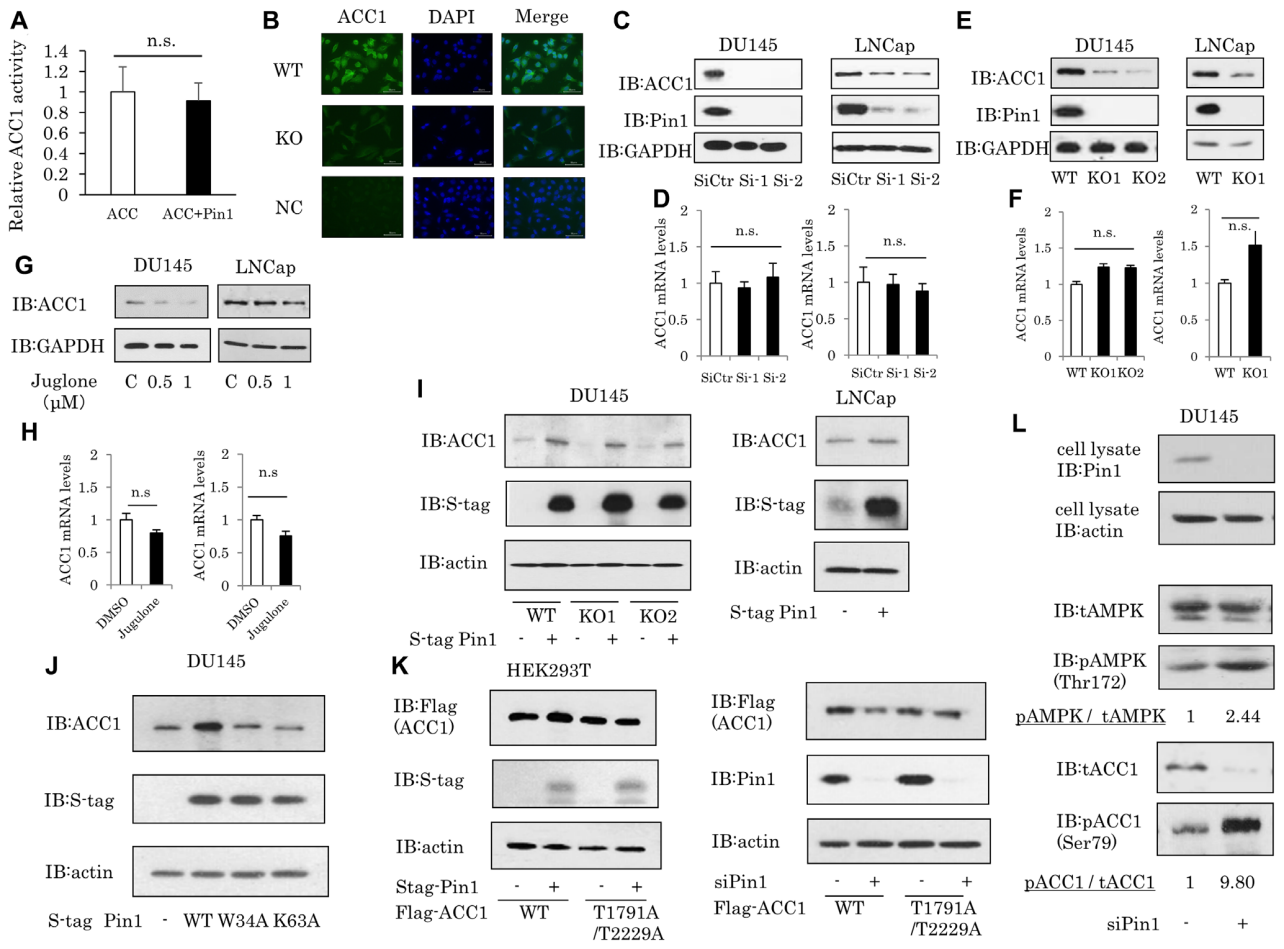
The Pin1 change parallels ACC1 changes (**A**) ACC1 activity *in vitro* was measured, using recombinant ACC1 and Pin1. (**B**) WT DU145 or Pin1-KO DU145 cells were subjected to immunostaining with ACC1 antibody. NC is the negative control. (Green: staining of ACC1, blue: DAPI) (**C**) DU145 or LNCap cells were treated with Pin1 siRNA for 48 hrs, and ACC1 protein levels were then examined by Western blotting. (**E**) Lysates were prepared from WT or Pin1-KO cells using the CRISPR/Cas9 system. ACC1 protein levels were examined by Western blotting. (**G**) The Pin1 inhibitor Juglone was applied to DU145 or LNCap cells for 24 hrs. (**D, F, H**) ACC1 mRNA levels were examined by real-time PCR. (*n* = 4) (**I**) ACC1 protein levels overexpressing Pin1 in DU145 and LNCap cells. (**J**) ACC1 protein overexpression in wild type Pin1 or Pin1 mutants of DU145 cells. (**K**) Left: Protein level of Flag-ACC1. Wild-type or mutant Flag-ACC1, in which two Pin1 binding sites (T1791, T2229) were substituted with Ala, was co-expressed with S-tag Pin1 in HEK-293T cells. Right: Protein level of Flag-ACC1. At 24 hrs after transfection with Pin1 siRNA, transfection of wild-type or mutant Flag-ACC1 was performed for 48 hrs in HEK-293T cells. (**L**) At 96 hrs after transfection with Pin1 siRNA, DU145 cells were treated with 1 mM 2-DG(2-deoxyglucose) for 1 h. The ratio of AMPK phosphorylation at Thr172 to ACC1 phosphorylation at Ser79 (phosphorylated AMPK / total AMPK) was calculated based on protein determinations. Protein amounts were quantified using Image J.

Since the C-terminal carboxyltransferase domain of ACC1, which is involved in transferring the carboxyl group to acetyl CoA, is responsible for associating with Pin1 (Figure 3), the possibility that Pin1 affected ACC1 activity was investigated, employing an *in vitro* ACC1 activity assay using recombinant ACC1 and Pin1 proteins. The activity of recombinant ACC1 was unaffected, by either the presence or the absence of Pin1 protein (Figure 4A).

Subsequently, since Pin1 reportedly alters the subcellular location of certain target proteins, particularly between the cytoplasm and nuclei, we prepared Pin1-KO DU145 cells using the CRISPR/Cas9 system and investigated whether Pin1 impacts the subcellular localization of ACC1. However, ACC1 was located in the cytoplasm but not in nuclei, regardless of the presence or absence of Pin1 (Figure 4B).

Furthermore, we examined whether Pin1 alters the cellular amount of ACC1 protein, by applying Pin1 siRNA treatment or Pin1 overexpression. Pin1 knockdown by siRNA was revealed to dramatically decrease ACC1 protein levels in both DU145 and LNCap cells (Figure 4C). Similarly, Pin1 gene deficiency also decreased the ACC1 protein expressions in two types of cells (Figure 4E). Moreover, treatment with the Pin1 inhibitor Juglone produced the same results (Figure 4G). Interestingly, altered Pin1 expression exerted no effects on ACC1 mRNA levels (Figure 4D, 4F, 4H).

Conversely, the effect of Pin1 overexpression on the ACC1 protein level was investigated. It was shown that Pin1 overexpression in wild-type or Pin1-deficient DU145 cells, as well as LNCap cells, markedly increased ACC1 protein levels (Figure 4I). In addition, the increase in ACC1 protein was induced by overexpression of wild-type Pin1, but not by WW domain mutant (W34A) or isomerase activity dead (K63A) Pin1 mutants (Figure 4J). Furthermore, Pin1 overexpression increased the co-overexpression of full-length ACC1, but not that of T1791A/T2229A mutated ACC1, which is unable to bind to Pin1 (left panel of Figure 4K). Similarly, treatment with Pin1 siRNA reduced the wild-type ACC1, but did not alter the level of T1791A/T2229A mutated ACC1 (right panel of Figure 4K).

We also investigated whether Pin1 impacts the level of ACC1 phosphorylation by AMPK, since Pin1 reportedly suppresses AMPK activity. [35] As expected, it was found that siRNA-mediated Pin1 reduction enhanced the level of phosphorylation of AMPK-Thr172 (middle panel of Figure 4L). Enhancement of the phosphorylation level of ACC1-Ser79, downstream of AMPK, was clearly observed, despite the reduced ACC1 protein amount (lower panel of Figure 4L).

### Pin1 promotes the stability of ACC1 protein

**Figure 5:**
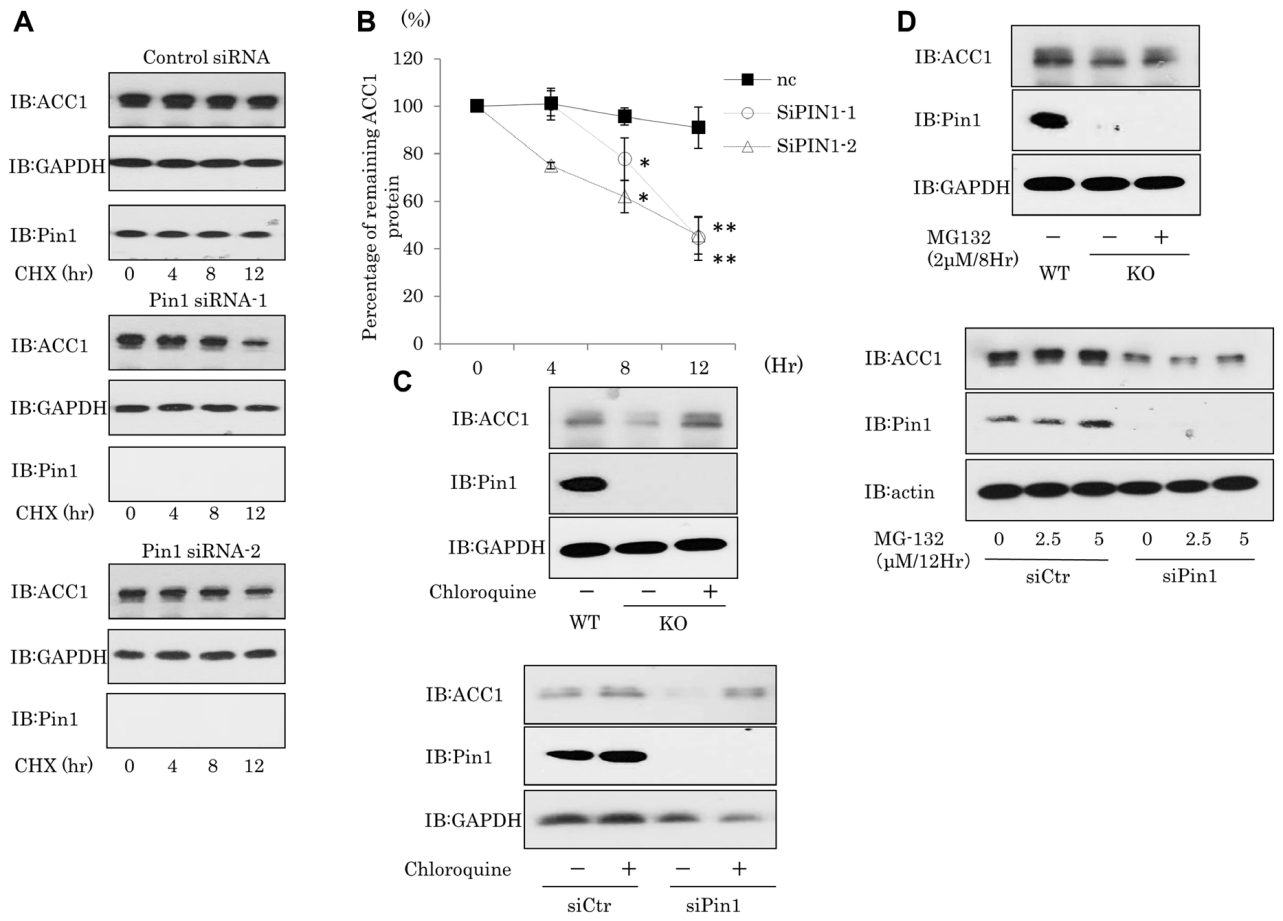
Pin1 suppresses ACC1 protein degradation (**A, B**) DU145 cells were treated with Pin1 siRNA. After 48 hrs, cycloheximide (20 μg/ml) was added to the culture medium for the indicated times. Protein amounts were quantified using Image J. (*n* = 3) (**C, D**) Cells were treated with 2 μM MG-132 for 8 hr or 100 μM chloroquine for 48 hrs.

To assess whether Pin1 inhibits the degradation of ACC1 protein and thereby increases ACC1, the half-life of ACC1 protein was examined employing experiments using DU145 cells and cycloheximide. ACCl protein was relatively stable with a half-life of about 24 hours in the untreated or control siRNA treated condition in DU145 cells (upper panel of Figure 5A, Figure 5B). In contrast, knockdown of Pin1 by siRNA treatment markedly shortened the half-life of ACC1 to less than 12 hours (middle and lower panels of Figure 5A, Figure 5B). These data suggest that Pin1 elavates the stability of ACCl. Given that Pin1 prevented the degradation of ACCl, we examined possible involvements of the lysosomal and proteasome pathways. As shown in Figure 5C, when Pin1 deficient or knockdown cells were treated with chloroquine, an inhibitor of the lysosomal pathway, ACC1 protein expression was increased, whereas the proteasome inhibitor MG-132 had no effect on the stability of ACC1 protein (Figure 5D). Taken together, these results indicate that Pin1 inhibits the degradation of ACC1 protein through the lysosomal pathway.

### Upregulation of the ACC1 expressions in human prostate cancer

**Figure 6:**
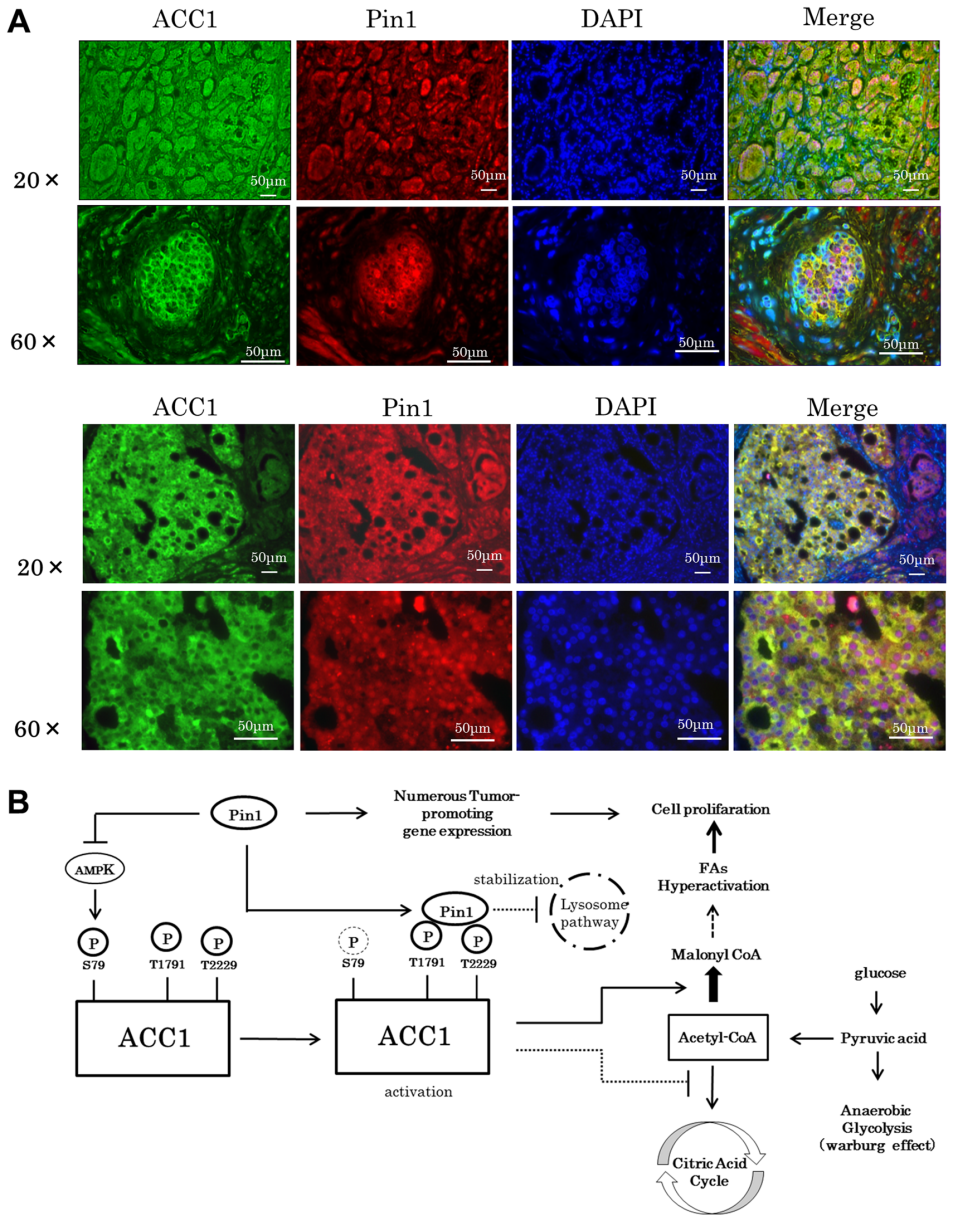
Both Pin1 and ACC1 expressions in human prostate cancers are upregulated (**A**) Sections of the prostate cancer specimens from two patients were immunostained with ACC1 and Pin1 antibody. The scale bar shown at the bottom of the figure is 50 μm (Green: ACC1, Red: Pin1, Blue: DAPI). (**B**) Proposed mechanism of prostate cancer induced via the Pin1-ACC1 pathway.

Finally, representative ACC1 and Pin1 immunostaining data of prostate cancer specimens from two patients are shown in Figure 6A. Very similar results were obtained when we examined cancerous parts of the prostate specimens obtained from the other 8 patients (data not shown). In stroma cells, expressions of both ACC1 and Pin1 were relatively low. Interestingly, the expressions of ACC1 were observed to be modestly increased in human prostate cancer cells. In addition, in these malignant cells, Pin1 expression levels were also upregulated, suggesting that Pin1 upregulates the expressions of ACC1 protein in human prostate cancers (Figure 6A).

## DISCUSSION

Pin1 plays a critical role in most cancers by promoting malignant features, such as cell proliferation, and is thereby associated with a poor prognosis [1–3]. To date, more than 30 proteins promoting growth and survival have been identified, the functions and/or stability of which are elevated by Pin1. In contrast, Pin1 reduces the activity and/or stability of 20–30 anti-oncogenic proteins [1, 34]. Notably, cancer cells possess different features in terms of metabolism and energy production, in comparison with normal cells. The most intensively studied issue is the involvement of Pin1 in the mechanism underlying the Warburg effect, as mentioned in the Introduction [8, 9, 36, 37]. In addition, increased *de novo* synthesis of FAs is also a distinctive metabolic feature of cancer cells [38–40]. Both ACC1 and ACC2 generate malonyl-CoA, though at different subcellular locations, as rate limiting enzymes of FA synthesis. Malonyl-CoA serves as the substrate for FA synthesis in the cytoplasm, and FAs are essential as both energy sources and in the formation of cell membranes [16, 41, 42].

In the ATP deficient condition in normal cells, ACC1 and ACC2 activities are suppressed by AMPK and acetyl CoA is utilized in the citric acid cycle. Although both isoforms of ACC are phosphorylated and inactivated by AMPK, AMPK-mediated inhibition of ACC2, but probably not of ACC1, appears to be involved in the activation of carnitine palmitoyltransferase-1 (CPT-1), which promotes FA oxidation in mitochondria [15, 43]. On the other hand, in most cancer cells, ACC1 protein is constitutively upregulated, resulting in increased lipogenesis and inhibition of the citric acid cycle. Generally, ATP production through the electron transport chain and lipogenesis take place alternately, as acetyl CoA is utilized in both pathways. When ATP production in normal cells is required, ACC1 expression is suppressed to low level and acetyl CoA is utilized in the citric acid cycle [6, 44]. In cancer tissues, however, it is suggested that increased Pin1 constitutively upregulates ACC1 protein, resulting in lipogenesis and inhibition of the citric acid cycle. This phenomenon may contribute to Warburg effects.

The importance of FA synthesis for cancer cell growth was confirmed by the data showing that pharmacologic or genetic inhibition of ACC1 suppressed the proliferation of prostate cancer cells (Figure 1A, 1B), which agrees well with the results of previous reports on other types of cancer cells. In addition, it was also reported that an allosteric inhibitor of ACC induced apoptosis *in vitro* and suppressed the growth of xenografted cancers in nude mice [45]. Based on the aforementioned findings, we focused on the interaction between Pin1 and lipid metabolism, and identified an association between Pin1 and ACC1. The ACC1 domain binding to Pin1 was identified as the C-terminal carboxyltransferase domain, but no effect of Pin1 on enzymatic activity was observed. Instead, Pin1 inhibits the degradation of ACC1 protein and thereby increases the ACC1 protein level, but not that of ACC2. Although the ACC1 mRNA level was reportedly upregulated transcriptionally in most cancer cells [8, 46], the results of this study revealed that an altered Pin1 expression level had no significant effect on ACC1 mRNA levels. Thus, it is very likely that the high expression of ACC1 protein in cancer cells is caused by two independent mechanisms; at the transcriptional level and at the Pin1-mediated posttranscriptional-level.

Additionally, it should be noted that Pin1 strongly inhibits phosphorylation in the gamma subunit of AMPK and its kinase activity by associating with the gamma-subunit of AMPK [35]. Inactivated AMPK leads to reduced Ser79 phosphorylation of ACC1 and thereby elevates ACC1 activity [15]. Therefore, the ACC1 activity per protein is apparently enhanced through suppression of AMPK activity in most cancers with increased Pin1 expression.

Nevertheless, it is reasonable to assume that Pin1-induced ACC stabilization has no relationship with Pin1-induced AMPK suppression. It is because the C-terminal domain of ACC1 is necessary for Pin1-induced ACC1 stabilization, while phosphorylated Ser79 in ACC1 is involved in ACC1 inactivation. Taken together, these observations indicate that Pin1 enhances ACC1 activity by both inhibiting its degradation and enhancing enzymatic activity, though via different mechanisms (Figure 6B). Consistent with these findings, Pin1 inhibitors have been shown to exert therapeutic effects against cancers. For example, all trans retinoic acid (ATRA) decreases Pin1 expression levels through promoting the degradation of Pin1 protein, thereby suppressing the proliferation of breast cancer cells [47]. Moreover, API-1 which inhibits PPIase activity also decreases the development of hepatocellular carcinoma through activating exportin-dependent miRNA transfer [48]. These overall findings support the possibility of Pin1 serving as a therapeutic target for cancer cells, as previous studies have suggested.

## MATERIALS AND METHODS

### Materials

Antibodies were purchased from Abcam (S-tag: GR124515-1), Sigma (Flag: F3040), Santa Cruz Biotechnology (Pin1: sc-271441, GAPDH: sc-47724, tubulin: sc-8035 and actin: sc-47778) or Cell Signaling Technology (ACC1: #4190, phospho-ACC1 (Ser79): #11818, ACC2: #8578, AMPK-alpha: #2532, and phosphor-AMPK-alpha (T172): #2535). Dulbecco’s modified Eagle’s medium (DMEM) was purchased from Nissui (Tokyo, Japan), SYBR Green from KAPA (Shiga, Japan).

### Plasmids

ACC1 or Pin1 plasmids were prepared, as follows. Briefly, cDNAs encoding human ACC1 or human Pin1 with S -tag at their N terminal, were inserted into each Flag-pcDNA or pcDNA3.1 (-). Mutants with one substitution were created by using a KOD mutagenesis kit (TOYOBO).

### Cell culture

DU145, LNCap and HEK-293T cells were cultured in DMEM containing glutamine, NaHCO_3_, antibiotics, and 10% fetal calf serum (FCS). In experiments to confirm the phosphorylations of AMPK and ACC1, DU145 cells were stimulated for 1 hour with 10 μM 2-DG (2-deoxyglucose) prior to cell lysate recovery.

### ACC1 activity assay

Human ACC1 was adjusted to 100 ng and recombinant Pin1 protein 1 μg to 100 μM ATP and 20 μM acetyl CoA. Cells were incubated at 37°C for 60 minutes. The finally generated ADP - Glo^R^ was measured with a luminescent assay.

### Immunohistochemical staining

Paraffin-embedded sections of human prostate cancer tissues were deparaffinized employing xylene and ethanol, and the slides were then incubated with 0.1% Triton for 5 min. After being washed with phosphate-buffered saline (PBS) three times, the samples were boiled in citrate buffer (pH = 6.0) to activate antigens. Sections were washed again and reacted with primary antibody (1:400) overnight at 4°C. Thereafter, sections were incubated with FITC or Cy3-conjugated secondary antibodies for 1 hr at room temperature. Finally, the slides were mounted with DAPI. Tissue samples used in this study were obtained from patients of Hiroshima University Hospital, and appropriate informed consent was obtained from each patient in accordance with the Ethical Guidance for Human Genome/Gene Research of the Japanese Government.

### Immunoprecipitation

Cultured cells were solubilized with lysis buffer containing 50 mM Tris-HCl, 150 mM NaCl, 1 mM EDTA, 1% Triton, 1 mM PMSF, 1 mM orthovanadate, and 1 mM NaF. After centrifugation at 15,000 rpm for 30 min, supernatants were transferred to new tubes. Adequate amounts of antibodies and beads were added, and the lysates were rotated for 2 h at 4°C. The beads were washed four times with lysis buffer. In the case of using Flag beads, immunoprecipitates were eluted employing Flag peptide. Finally, 2× SDS sample buffer was added to each tube, followed by boiling at 95°C for 5 min.

### GST pull-down assay

Flag-WT ACC1 or ACC1 mutants were overexpressed in HEK-293T cells. Then, cell lysates were immunoprecipitated employing Flag beads and Flag-ACC1 was eluted with the Flag peptide. Elutions containing Flag-ACC1 were mixed with GST or GST-Pin1 for 2 hr at 4°C. Finally, the beads were washed with lysis buffer four times and sample buffer was added.

### Immunoblotting

Proteins were subjected to SDS-PAGE and then transferred to PVDF membranes. After blocking with 3% bovine serum albumin in PBS/Tween for 1 h, the membranes were reacted with the first antibody (1:3000) overnight at 4°C, followed by the secondary antibody (1:4000) for 1 h at room temperature. The bands were detected using Super Signal West Pico stable peroxidase solution (Thermo).

### Gene silencing of Pin1 using siRNAs

DU145, LNCap and HEK-293T cells were transfected with either negative siRNA (Qiagen) or Pin1 siRNA (Invitrogen) using RNAiMAX (Invitrogen) according to the manufacturer’s protocol: Pin1 siRNA1, CCG UGU UCA CGG AUU CCG GCA UCC A; Pin1 siRNA2, GCC CUG GAG CUG AUC AAC GGC UAC A; ACC1 siRNA1, GGG ACU UCA UGA AUU UGC UGA UUC U; ACC1 siRNA2, CCU UAC AAG GGA UAC AGG UAU UUA U.

### Gene knockout of Pin1 using CRISPR/Cas9

To achieve Pin1 gene knockout in DU145 cells, both the Pin1 Crispr/Casp plasmid and the Pin1 HFD plasmid were transfected into DU145 cells. After two days, cells were exposed to 5 μg/ml puromycin for selection. The cells were then seeded into a 96-well plate for limiting dilution analysis. Pin1 knockout was confirmed by immunoblotting. We used pre-designed Crispr/Cas9 (sc-400485) purchased from Santa Cruz. This commercial Crispr/Cas9 contains three different gRNA pools and the sequences are as follows: AAGCGCATGAGCCGCAGCTC: GATGAGCGGGCCCGTGTTCA: AAGACGCCTCGTTTGCGCTG.

### Real-time PCR

RNA extraction was performed using Sepasol according to the manufacturer’s protocol. To measure the mRNA levels of ACC1 and GADPH, reverse transcription and real-time PCR were conducted as described previously (52). Primer sets were as follows: ACC-1 atgtggtggtctactctgatgtcaatc (forward) and ctacgtggaaggggaatccat (reverse), GAPDH ggcaccgcaggccccgggatgctagtg (forward) and tgatggcaacaatatccactttacc (reverse).

### MTT cell proliferation assay as a measure of cell viability and proliferation *in vitro*

Yellow tetrazolium MTT (3- (4,5-dimethylthiazolyl-2)5-diphenyltetrazolium bromide) was added to the cell culture medium to achieve a concentration of 0.25 mg/ml, followed by incubation at 37°C for 3 hours. Thereafter, the medium was removed and dissolved in isopropanol, and the absorbance at 570 nm was measured.

### Immunofluorescence staining

DU145 cells were grown on coverslips, fixed for 10 min in 2% formaldehyde, and permeabilized for 15 min in PBS containing 0.2% Triton X-100. Cells were then rinsed with PBS and blocked for 30 min at room temperature in PBS containing 0.2% Triton X-100 and 3% fetal bovine serum. Cells were then incubated with the anti-ACC1 (1:200) or anti-PIN1 (1:200) antibodies overnight at 4°C. After being washed, FITC-conjugated anti-rabbit (1:200) or Cy3 anti-mouse secondary antibodies (1:200) were added for 1 hr at room temperature. DNA was stained with 4,6- diamidino-2-phenylindole (DAPI) (5 µg/ml). Cells were mounted and images were captured with a wide-field Fluorescence Microscope (BZ-9000E / KEYENCE Inc.).

### Statistical analysis

Data are expressed as means ± S.E., and values of *P* < 0.05 were considered to be statistically significant. (^*^P < 0.05, ^**^P < 0.01,^***^P < 0.001).
